# The Spectrum of ACS: Towards a More Personalized Approach

**DOI:** 10.3390/life11040322

**Published:** 2021-04-06

**Authors:** Thomas F. Lüscher

**Affiliations:** 1 Royal Brompton & Harefield Hospitals, National Heart and Lung Institute, Heart Division and Imperial College, London SW3 6LY, UK; cardio@tomluescher.ch; Tel.: +44-7502-008-487; 2 Center for Molecular Cardiology, University of Zurich, 8952 Schlieren, Switzerland

**Keywords:** plaque rupture, erosion, coronary dissection, Takotsubo syndrome, myocarditis

## 1. How It All Started

On 24 September 1955, Wall Street was in a panic and shares plummeted. Eventually, the Dow Jones had lost 6% or $14 billion, and for good reason; the day before, the president of the USA, Dwight D. Eisenhower, had a heart attack and was hospitalized for that reason. It happened while he was playing golf at Cherry Hills Country Club when Ike, as the Americans called him, complained of indigestion [1]. The President’s personal physician, Dr. Howard Snyder, obviously not an expert in heart disease, considered it a gastroenteritis and waited 10 h before sending the President to the next hospital. At last, he treated Eisenhower with amyl nitrate for his angina and morphine against the increasingly heavy pain. Consequently, Eisenhower fell into a deep sleep until the next morning. The chest pain persisted and finally an electrocardiograph was brought from another hospital that recorded an anterolateral acute myocardial infarction with ST segment elevations across the anterior leads; obviously, this was not what we would consider today guideline-based management of myocardial infarction [2].

**Figure 1:**
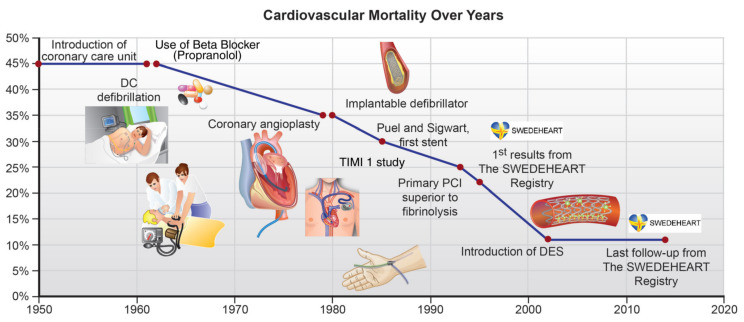
Impact of major advances in cardiovascular care on mortality (reproduced from [2] with permission)**.**

We have made progress since Eisenhower’s heart attack and it is impressive indeed (Figure 1): just shortly after that, Paul M. Zoll from Harvard developed defibrillation to treat ventricular fibrillation [3], a then common and fatal complication of myocardial infarction. Then, betablockers were developed by Sir James Black [4], and coronary angiography by Mason Soanes; Sir John Vane discovered the platelet inhibitory effects of aspirin [5], Grüntzig performed his seminal first balloon angioplasty of a coronary stenosis [6], and Peter Sleight published the ISIS-2 trial showing the protective effects of aspirin and streptokinase [7]. Primary angioplasty was introduced [8], followed by the development of coronary stents, then P_2_Y_12_-receptor blockers such as, initially, ticlopidine, clopidogrel and now prasugrel and ticagrelor, followed by statins [9] and the radial approach for primary percutaneous coronary intervention (pPCI). Mortality improved from 50% (for those who even reached a hospital in the 1950s) to currently 10% [2] (Figure 1). More recently, for the secondary prevention of hyperlipidemia, the Niemann–Pick transport inhibitor ezetimibe, and inhibitors of the proprotein convertase subtilisin/kexin type 9 (PCSK9) have become available and allow for even further reductions in LDL cholesterol in high-risk patients.

To blunt left ventricular remodeling, angiotensin converting enzyme inhibitors have been shown to be effective [10], eventually reducing heart failure after an acute coronary syndrome. While betablockers are still widely prescribed, the evidence for their protection from sudden cardiac death stems from the pre-PCI era [11]; whether they remain essential with today’s guideline-based management with primary PCI is uncertain.

## 2. STEMI and NSTEMI

**Figure 2:**
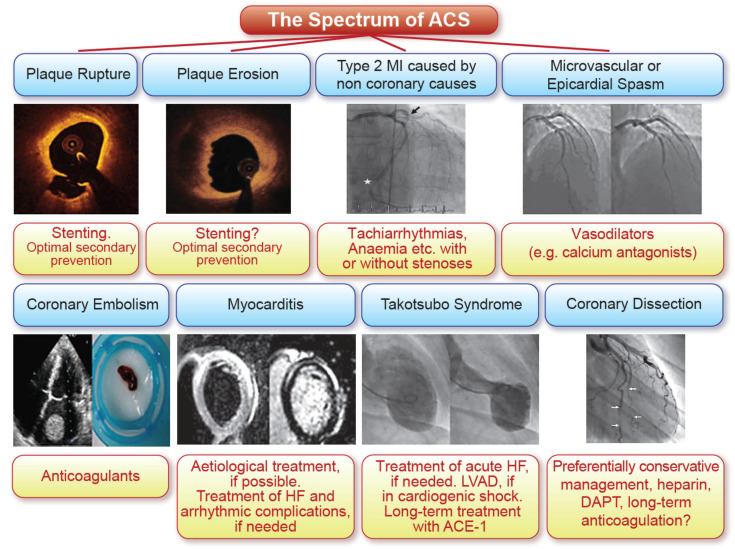
The spectrum of acute coronary syndromes (ACS) from reference [15] with permission.

In Eisenhower’s time, there was only one myocardial infarction: the heart attack. Much later, when thrombolysis was introduced, it was noticed that it only worked in patients with ST-segment elevations on the ECG, and splitting began. Heart attacks were divided into ST-segment elevation myocardial infarction (STEMI), non-STEMI, unstable angina, and later also into type 1 to 5 infarctions [12] which all required, as it turned out, a different management. As a consequence, today, separate guidelines for STEMI [13] and NSTEMI [14] are published by the *European Heart Journal* and the spectrum of acute myocardial infraction is beginning to expand (Figure 2).

## 3. Plaque Rupture and Erosion

For many decades, it remained unclear how a major coronary artery occludes and causes infarction until Michael Davies introduced the concept of plaque rupture [16]. A large body of experimental evidence then provided the understanding of how an atherosclerotic plaque may rupture. It has been known from the original pathological studies that rupture mainly occurs at the shoulder of the plaque [17]. Later, it became clear that the fibrous cap covering an atherosclerotic plaque, consisting mainly of collagen, is weakened by inflammation [18,19,20] via activation of Toll-like receptor-4 [21] and eventually digested by enzymes, such as metalloproteases and fibrin activation peptide [22], among others. The exposure of these subendothelial structures markedly activates circulating platelets which form an evolving clot. Thus, rupture of a vulnerable plaque occurs either by specific triggers such as hemodynamic factors (i.e., acute increase in shear stress and/or pressure), coronary spasm, inflammatory bursts, or intraplaque bleeding from the vasa vasorum.

The bigger the exposed tissue and/or cavity of a plaque rupture, the more intensively the circulating platelets are activated and the bigger the involving thrombus that eventually forms. In the context of activation of the coagulation cascade with fibrin formation, an occluding thrombus may form. Indeed, patients with ruptured plaques on optical coherence tomography (OCT) have a worse outcome compared to those with intact fibrous caps [23]. Of note, the more fibrin an evolving thrombus contains, the more solid the thrombus will be. Thrombus solidity has been recognized now as an independent risk factor for further events in patients with ST-segment elevation myocardial infarction (STEMI) [24]. The activity of endogenous fibrinolysis determines whether or not an evolving thrombus may again be dissolved spontaneously, and plaque healing may occur. The more pronounced the endogenous fibrinolysis system, the more likely the patient experiences transient STEMI with spontaneous re-establishment of coronary blood flow after some time [25]. Such STEMIs have a much better prognosis than those of patients with reduced endogenous fibrinolysis and highly solid thrombi. Coronary embolism in patients with a patent foramen ovale (atrial fibrillation or left ventricular thrombi) is quite a rare event and often difficult to adjudicate.

More recently, it has been recognized that besides plaque rupture, erosion may also cause acute coronary syndromes, be it STEMI or non-STEMI, respectively [26]. It has been estimated that plaque erosion may account for around 25–40% of acute coronary syndromes (ACS), predominantly affecting smokers, women and younger patients. Still today, the diagnosis remains one of exclusion of plaque rupture in patients with obstructive lesions, typically using OCT as an intravascular imaging modality [27]. The mechanisms leading to plaque erosions are less clear. Histopathological and experimental data suggest that endothelial denudation as well as extracellular matrix (ECM) degradation are key features of plaque erosion [28]. Recently, an important role for the local cytotoxic response mediated by CD8 T lymphocytes in plaque erosion occurring in the presence of a local alteration of shear stress has been suggested as a major mechanism [29].

STEMIs due to plaque erosion may present after the opening of an occluded coronary artery with a guide wire and/or the balloon, often as hemodynamically non-obstructive lesions. OCT studies have suggested that under these conditions with a proven absence of plaque rupture, patients may be treated with a balloon only without a stent and aggressive secondary prevention, in particular, dual antiplatelet therapy and statins [30]. This concept of a more personalized approach to patients with STEMI, however, needs further evaluation in large, randomized trials.

## 4. Other Types of Infarction

The diagnosis of myocardial infarction started with a clinical impression and the ECG; then, creatinine kinase was the golden standard. Today, troponin is the primary biomarker for the diagnosis [31]. *The Universal Definition of Myocardial Infarction* distinguishes five types of infarction: (1) Type 1: acute coronary occlusion due to plaque rupture, erosion or embolism; (2) Type 2: acute ischemia due to an imbalance of supply and demand due to hypotension or severe hypertension, bleeding and/or fast heart rate, including epicardial vasospasm; (3) Type 3: sudden cardiac death with suspicion of ischemia as the underlying cause; (4) Type 4: peri-interventional infarction after PCI, commonly due to side branch occlusion; and (5) Type 5: perioperative infarction during bypass surgery [12].

## 5. MINOCA

Surprisingly for many operators, some patients presenting with typical chest pain, ST-segment changes and/or T-inversions and elevated troponin levels may present at angiography with non-obstructive coronary arteries [32]. This condition has been named *Myocardial Infarction with Non-Obstructive Coronary Arteries* or MINOCA. What is behind this presentation? Indeed, MINOCA may be caused by a number of conditions. First, patients with transient STEMI (see above) may present as MINOCA. Indeed, patients with a highly effective endogenous fibrinolysis and ST-elevations when presenting to the emergency room or to paramedics sometimes show normalization of the ECG and open coronary arteries once they arrive in the catheterization laboratory. This may particularly be the case in those with endothelial erosion rather than plaque rupture. Furthermore, the underlying plaque rupture may not be visible, angiographically, for a variety of reasons or may have been overlooked. Coronary spam is probably rare in Caucasians but is a common cause in Japan and possibly other Asian countries. Finally, microvascular dysfunction has been suspected as a possible cause of MINOCA. Indeed, some investigators have suggested using provocative tests with acetylcholine or ergonovine to prove macrovascular or microvascular spasm [33].

Of note, the outcome of patients with MINOCA is far from excellent. Indeed, such patients have an important rate of major cardiovascular events (MACE) after such an acute event, albeit somewhat less so than those with obstructed coronary arteries and infarction [34]. Accordingly, while such patients should not undergo primary PCI, they should receive the same aggressive secondary preventions as STEMI or NSTEMI patients, respectively.

## 6. Spontaneous Coronary Dissection

The story goes on; spontaneous coronary dissection (SCAD) is due to an acute development of a false lumen within any coronary artery, which compromises coronary flow by compression of the true lumen leading to myocardial ischemia and symptoms of acute chest pain. Iatrogenic dissections due to catheter manipulations within the coronary system do not fall under this definition. Thus, SCAD is another, altogether quite different, acute coronary condition. While initially considered a rare condition affecting primarily pregnant women, it is now recognized that SCAD is a not uncommon form of ACS affecting 2–4% of all patients undergoing angiography (predominantly young to middle-aged women, commonly occurring outside the context of a recent pregnancy) [35]. SCAD has been associated with the *PHACTR1/EDN1* genetic locus, thus possibly genetic factors may be essential, while acute triggers of the condition may involve a number of hemodynamic factors, inflammation and/or spasm.

The diagnosis of SCAD is challenging as it is clinically, on ECG and based on biomarkers, currently indistinguishable from NSTEMI or STEMI, respectively. Thus, the diagnosis of SCAD requires coronary angiography and often intravascular imaging such as OCT. SCAD has been classified based on different angiographic presentations. Type 1 represents the classical angiographic radiolucent “flap” and linear double lumen often associated with contrast hold-up, while the more common Type 2 is characterized by a long diffuse and smooth stenosis predominantly located in mid-to-distal coronary segments. The rare Type 3 lesions are angiographically indistinguishable from a focal coronary stenosis requiring intracoronary imaging. Type 4 has led to a total occlusion, usually of a distal vessel which makes the diagnosis challenging.

The management of SCAD is no less challenging than the diagnosis. Indeed, PCI may cause total occlusion of the affected vessel, if the guidewire is inadvertently positioned in the false lumen. Thus, although randomized trials are missing, it appears based on registry data that a conservative approach with antiplatelet agents and/or anticoagulation might be more promising. Indeed, if the patient is pain free, conservative management reveals quite good results [36]. However, with ongoing ischemia and pain, a catheter-based intervention has to be considered.

## 7. Takotsubo Syndrome

Takotsubo syndrome (TTS) was first described by Sato et al., in 1990 in a 64-year-old female who presented with acute chest pain, typical ECG changes of a STEMI, but unobstructed coronary arteries and an unusual appearance of the left ventricle with a narrow neck and apical ballooning during systole. Surprisingly, the massive left ventricular dysfunction disappeared after two weeks [37]. While it was first assumed that TTS would only affect patients of Asian descent, it is now clear that TTS accounts for 2–4% of all patients transferred to the catheterization laboratory with suspected ACS. Importantly, TTS is not a harmless disease, but has an in-hospital mortality of around 4–5% with up to 15% presenting in cardiogenic shock [38]. The outcome also depends on the specific form of TTS with apical ballooning having the worst outcome, while basal and mid-ventricular forms have a much more favorable outcome.

The management of TTS is challenging; indeed, in many cases, tender loving care may suffice, while in others acute cardiac care is mandatory. Of note, the use of inotropes may be detrimental in those presenting with hypotension and shock, particularly in those presenting with apical ballooning as such drugs may induce a pressure gradient along the basal left ventricle and the outflow tract thereby deteriorating the hemodynamic conditions [39]. In such patients, assist devices such as the Impella or extracorporeal membrane oxygenation (ECMO) have to be considered.

## 8. Myocarditis

Myocarditis is an inflammation of the myocardium, which may have a fulminant, acute, subacute, or chronic clinical trajectory. Inflammatory changes in the myocardium, either induced by viruses and rarely by bacteria, or by an autoimmune reaction, commonly involve also the pericardium (i.e., peri-myocarditis). Under such conditions, patients experience chest pain and may present in the emergency department with ECG changes and increases in troponin levels making the differential diagnosis to an ACS in the proper sense challenging [40]. Acute myocarditis may also present with wall motion abnormalities up to frank cardiogenic shock. Indeed, such presentations have recently been also described in patients with Covid-19 infection.

Fulminant myocarditis is defined as a myocarditis that leads within four weeks or less of symptom onset to severe heart failure, requiring medical and/or mechanical cardiovascular support. The most common etiology is viral or autoimmune in nature and, less commonly, hypersensitivity and toxic reactions to drugs [41], as well as giant cell myocarditis (a rare but very aggressive form of myocarditis often associated with autoimmune diseases).

The diagnosis of myocarditis is commonly made based on clinical criteria and, more recently, using MRI revealing typical epicardial late enhancement and signs of inflammation and edema formation [42]. Eventually, however the diagnosis must rely on histological and molecular findings within myocardial biopsies.

In patients with mild forms and maintained left ventricular function, non-steroidal antinflammatory drugs are used for pain management [43], while those with pump failure are treated with heart failure drugs (i.e., ACE-inhibitors, betablockers, mineralocorticoid receptor antagonists) and, if required, antiarrhythmics, such as amiodarone. Patients in cardiogenic shock do require acute cardiac care up to ventilation and the use of assist devices or ECMO in some severe cases.

## 9. The Bottom Line

Thus, the spectrum of acute coronary syndromes has grown and an increasingly personalized management of these patients provides better and better outcomes (Figure 2) [2].

Of note, while patients with STEMI and non-STEMI should undergo primary or urgent PCI, respectively, and must receive aggressive secondary prevention focusing on their cardiovascular risk factors, patients with MINOCA should, obviously, only receive the latter. There is an increasing discussion on whether patients with plaque erosion and hemodynamically non-obstructive lesions, after removal of the occluding thrombus, should receive a stent or only aggressive secondary prevention. Patients with Takotsubo, on the other hand, should receive primarily tender loving care until their left ventricular function normalizes within days or weeks, except for patients presenting after cardio-pulmonary resuscitation, syncope or cardiogenic shock, for which acute cardiac care management is required. Importantly, patients with typical ballooning should not receive inotropes, but rather only vasoconstrictors (i.e., norepinephrine); those with ongoing and severe hypotension should be considered for ECMO or Impella treatment. Finally, patients with myocarditis most commonly only require treatment of pain in the case of peri-myocarditis, which is the most common presentation. This is commonly achieved with non-steroidal anti-inflammatory drugs (NSAIDs) or other pain medication. Patients with more severe forms of myocarditis and scar formation in the myocardium or impaired left ventricular function, or even cardiogenic shock, do require appropriate management with acute cardiac care up to ECMO and intubation with ventilation. In these cases, myocardial biopsy should be obtained. After the acute event, depending on the degree of myocardial bruises and scar formation and the occurrence of fatal arrhythmias, an implantable cardioverter defibrillator may be considered.

Thus, the bottom line of this viewpoint is that patients presenting with suspected acute coronary syndromes may have a variety of underlying conditions, which today require a personalized management tailored to their symptoms, clinical presentation and underlying causes.

## Data Availability

Not applicable.
